# Tomato Yellow Leaf Curl Virus-Resistant and -Susceptible Tomato Genotypes Similarly Impact the Virus Population Genetics

**DOI:** 10.3389/fpls.2020.599697

**Published:** 2020-12-07

**Authors:** Wendy G. Marchant, Saurabh Gautam, Samuel F. Hutton, Rajagopalbabu Srinivasan

**Affiliations:** ^1^Department of Entomology, University of Georgia, Tifton, GA, United States; ^2^Department of Entomology, University of Georgia, Griffin, GA, United States; ^3^Horticulture Sciences Department, University of Florida, Wimauma, FL, United States

**Keywords:** whitefly, tomato, TYLCV, host resistance, selection

## Abstract

*Tomato yellow leaf curl virus* is a species in the genus *Begomovirus* and family *Geminiviridae*. Tomato yellow leaf curl virus (TYLCV) infection induces severe symptoms on tomato plants and causes serious yield losses worldwide. TYLCV is persistently transmitted by the sweetpotato whitefly, *Bemisia tabaci* (Gennadius). Cultivars and hybrids with a single or few genes conferring resistance against TYLCV are often planted to mitigate TYLCV-induced losses. These resistant genotypes (cultivars or hybrids) are not immune to TYLCV. They typically develop systemic infection, display mild symptoms, and produce more marketable tomatoes than susceptible genotypes under TYLCV pressure. In several pathosystems, extensive use of resistant cultivars with single dominant resistance-conferring gene has led to intense selection pressure on the virus, development of highly virulent strains, and resistance breakdown. This study assessed differences in TYLCV genomes isolated from susceptible and resistant genotypes in Florida and Georgia. Phylogenetic analyses indicated that Florida and Georgia isolates were distinct from each other. Population genetics analyses with genomes field-collected from resistant and susceptible genotypes from Florida and/or Georgia provided no evidence of a genetic structure between the resistant and susceptible genotypes. No codons in TYLCV genomes from TYLCV-resistant or susceptible genotypes were under positive selection, suggesting that highly virulent or resistance-breaking TYLCV strains might not be common in tomato farmscapes in Florida and Georgia. With TYLCV-resistant genotypes usage increasing recently and multiple tomato crops being planted during a calendar year, host resistance-induced selection pressure on the virus remains a critical issue. To address the same, a greenhouse selection experiment with one TYLCV-resistant and susceptible genotype was conducted. Each genotype was challenged with TYLCV through whitefly-mediated transmission serially 10 times (T_1_-T_10_). Population genetics parameters at the genome level were assessed at T_1_, T_5_, and T_10_. Results indicated that genomes from resistant and susceptible genotypes did not differentiate with increasing transmission number, no specific mutations were repeatedly observed, and no positive selection was detected. These results reiterate that resistance in tomato might not be exerting selection pressure against TYLCV to facilitate development of resistance-breaking strains. TYLCV populations rather seem to be shaped by purifying selection and/or population expansion.

## Introduction

Tomato yellow leaf curl virus (TYLCV) infects tomato and causes substantial yield losses in the southeastern United States and in many parts of the world (Czosnek and Laterrot, 1997; Momol et al., 1999; Polston et al., 1999; Moriones and Navas-Castillo, 2000; Pappu et al., 2000; Varma and Malathi, 2003). Symptoms of TYLCV infection in tomato plants include stunted growth, chlorosis, curling of leaves, and reduced fruit yield (Cohen and Nitzany, 1966; Cohen and Antignus, 1994; Picó et al., 1996). *Tomato yellow leaf curl virus* is a species in the genus *Begomovirus* and in the family *Geminiviridae*. TYLCV is a monopartite DNA virus with circular genome that contains six genes with two genes on the viral strand (V1–V2) and four genes on the complementary sense strand (C1–C4) (Gronenborn, 2007). The virus is phloem limited in its hosts, and it is transmitted exclusively by the sweetpotato whitefly, *Bemisia tabaci* (Gennadius), in a persistent and circulative manner (Cohen and Harpaz, 1964; Cohen and Nitzany, 1966; Ghanim and Medina, 2007; Czosnek, 2008).

**Figure 1:**
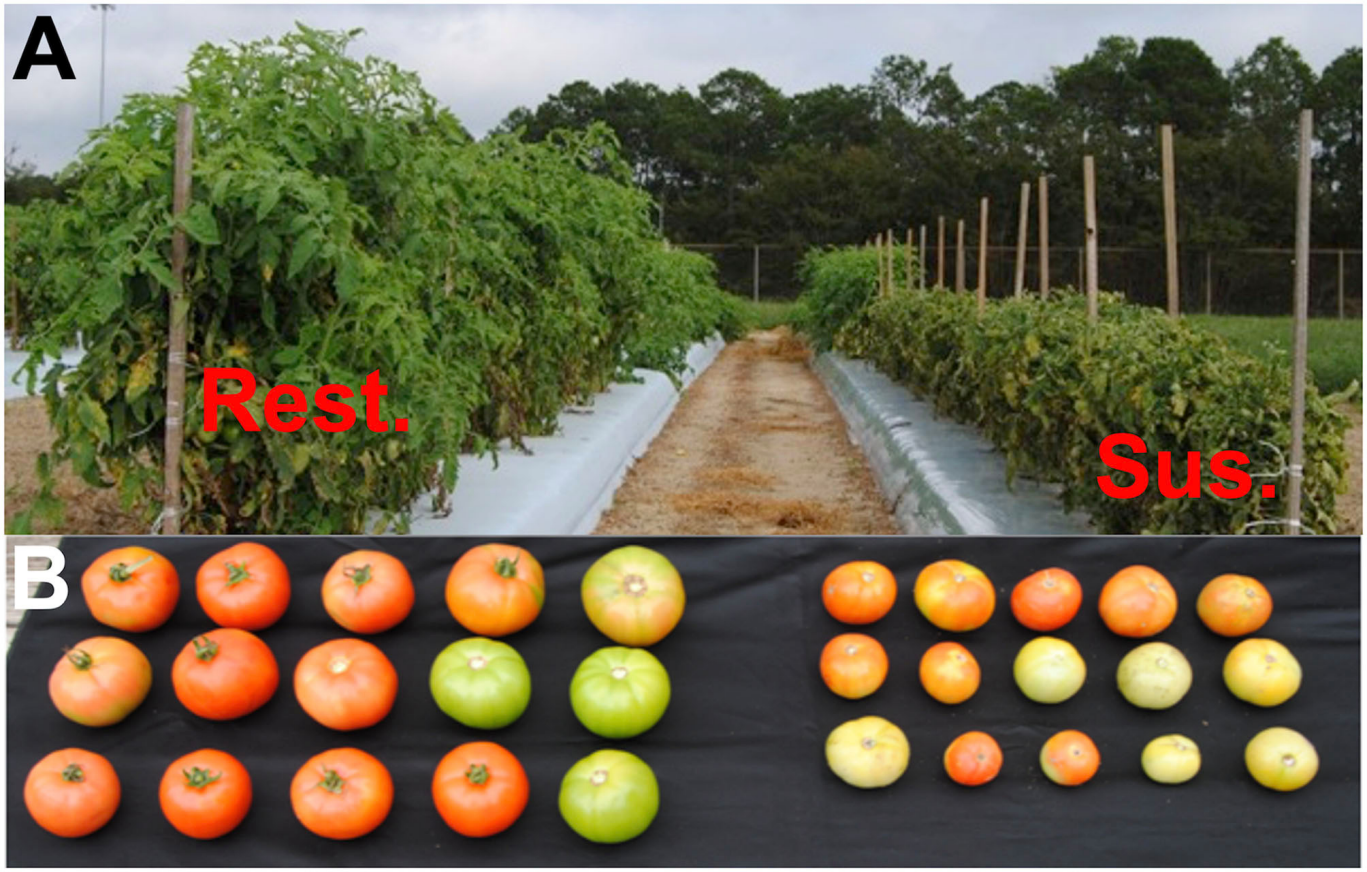
**(A)** Photograph representing differences in TYLCV infection symptoms on a TYLCV-resistant and -susceptible tomato genotype under intense TYLCV pressure. Both genotypes were planted at the same time. Photograph was taken ~2 months after planting. **(B)** Photograph representing typical size and quality of tomato fruits obtained from TYLCV-resistant (left) and -susceptible (right) genotypes.

Resistance to TYLCV has been incorporated from wild solanum species into tetraploid cultivated tomato (Lapidot et al., 1997; Lapidot and Friedmann, 2002; Yan et al., 2018). TYLCV-resistant tomato cultivars and hybrids (hereafter referred to as genotypes) have proven to be effective in managing the virus (Lapidot et al., 1997; Gilreath et al., 2000; Lapidot and Friedmann, 2002). TYLCV-resistant tomato genotypes are not immune to the virus, and do not completely stop the replication of TYLCV. TYLCV-resistant genotypes are systemically infected, often exhibit milder symptoms, and suffer reduced yield loss than susceptible genotypes (Figure 1). Resistant genotypes also accumulate lower levels of the virus compared with susceptible genotypes (Lapidot et al., 2001; Legarrea et al., 2015). Consequently, whiteflies acquire reduced amounts of virus from TYLCV-infected resistant genotypes than susceptible genotypes, suggesting that resistant genotypes might not function as effective inoculum sources in comparison with susceptible genotypes (Lapidot et al., 2001; Legarrea et al., 2015). Resistance to TYLCV was initiated largely by exploitation of the *Ty*-1 semi-dominant gene (Zamir et al., 1994). In addition to *Ty*-1 gene, more recently developed resistant stocks contain *Ty*-2, *Ty*-3, *Ty*-4, *ty*-5, and *Ty*-6 genes (Hanson et al., 2000; Ji et al., 2007, 2009; Anbinder et al., 2009; Hutton et al., 2012). Recent studies have shed light on mechanisms of resistance for these resistance-conferring genes. *Ty-*1and *Ty-*3 have been identified as RNA-dependent RNA polymerases (Verlaan et al., 2013). *Ty-*2 has been identified as a nucleotide binding domain containing leucine rich repeat gene (NB-LRR) (Yamaguchi et al., 2018). *Ty-*4 has been identified as a partial dominant gene (Ji et al., 2009; Kadirvel et al., 2013), and the *ty-*5 gene encodes a mRNA surveillance factor Pelota (Lapidot et al., 2015). Most recently, *Ty*-6 has been characterized as the incomplete dominance gene (Gill et al., 2019).

TYLCV-resistant genotypes were not initially preferred in the southeastern United States due to the reduced fruit size, poor taste, and non-uniform ripening qualities (Ozores-Hampton et al., 2010; Srinivasan et al., 2012). However, horticultural traits have substantially improved in recently available TYLCV genotypes, and resistant genotypes are increasingly planted (Riley and Srinivasan, 2019). Tomato also is grown nearly year-round in Florida and Georgia, thereby providing numerous opportunities for positive selection against TYLCV. TYLCV mutates at a very high rate. The rate of mutation for the full-length TYLCV genome is 2.88 × 10^−4^ substitutions/site/year, and it is comparable to RNA viruses (Duffy and Holmes, 2008). This high mutation rate combined with selection pressure from continued use of resistant cultivars could lead to the emergence of resistance-breaking TYLCV strains.

Numerous examples of resistance-breaking virus strains have been documented in other pathosystems. Beet necrotic yellow vein virus (BNYVV), an RNA virus, has overcome the *Rz1*-resistance gene in sugar beet in northwestern Europe due to mutations in the pathogenicity gene P25 (Bornemann et al., 2015). Tomato spotted wilt orthotospovirus (TSWV), also an RNA virus, has overcome resistance genes *Tsw* and *Sw5* in pepper and tomato, respectively. *Tsw* and *Sw5* resistance breakdown has been documented in Asia, Europe, and in the Americas (Latham and Jones, 1998; Roggero et al., 2002; Aramburu and Marti, 2003; Ciuffo et al., 2005; Sharman and Persley, 2006; Deligoz et al., 2014; Almási et al., 2015; Debreczeni et al., 2015; Ferrand et al., 2015; Jiang et al., 2016; Batuman et al., 2017). Another RNA virus, cotton leafroll dwarf virus (CLRDV), in Brazil overcame resistance in cotton accessions that were originally resistant (da Silva et al., 2015).

No instances of TYLCV overcoming *Ty*-induced resistance through positive selection in the field have been documented yet. However, an experiment in the lab led to the development of a resistance-breaking strain of TYLCV (Ohnishi et al., 2016). The tomato cultivar H24 was homozygous for the *Ty-*2 gene, and it was resistant to the TYLCV-IL strain but not the TYLCV-Mld strain. A virus chimera created in lab with the C4 and C1 genes from the Mld strain and the remainder of the genome from the IL strain led to resistance breakdown (Ohnishi et al., 2016). Similarly, if a natural recombination event occurred, resistance-breaking TYLCV strains could emerge under field situations. This was witnessed in southern Morocco with the recombinant TYLCV-IS76 that outcompeted its parents TYLCV-IL and tomato yellow leaf curl Sardinia virus (TYLCSV-ES) in tomato genotypes with the *Ty*-1 resistance gene (Belabess et al., 2016). This recombinant is now the prevalent TYLCV present in the region. Extensive use of TYLCV-resistant cultivars can also displace certain begomoviruses and/or their strains in favor of others. A survey in Spain found that susceptible genotypes in tomato fields were more often infected with TYLCSV-ES, while resistant genotypes with the *Ty-*1 gene in tomato fields were more often infected with TYLCV (García-Andrés et al., 2009).

This study attempted to examine if there is evidence for continuous use of TYLCV resistant cultivars resulting in TYLCV overcoming resistance and/or affecting TYLCV diversity in Florida and Georgia in southeastern United States. This was accomplished by examining the full-length genomes of naturally occurring TYLCV isolates from TYLCV-resistant and susceptible tomato genotypes from Florida and Georgia. Through a simulated greenhouse experiment, this study also attempted to determine if whitefly-mediated serial transmission of TYLCV involving a resistant genotype would lead to increased selection on the virus and development of resistance-breaking strains.

## Materials and Methods

### Maintenance of Whiteflies and TYLCV

The sweetpotato whitefly, *B. tabaci* Middle East-Asia Minor 1 (MEAM1) cryptic species (GenBank accession number MN970031), was first collected in Tifton, Georgia, USA in 2009. The whiteflies since then were maintained on 15 to 20 cm tall cotton plants in 45L × 45W × 90H cm^3^ whitefly-proof cages (Megaview Science Co., Taichung, Taiwan) in a greenhouse at 25–30°C with a 14 h L:10 h D photoperiod. The TYLCV isolate (GenBank accession number KY965880) was collected from a TYLCV-infected tomato plant in Montezuma, Georgia, USA in 2009. The virus has since been maintained in tomato (cultivar Florida 47, Seminis Vegetable Seeds, MO, USA) through whitefly-mediated transmission in the greenhouse at above-stated conditions.

### Isolation of TYLCV From Field-Collected TYLCV-Resistant and -Susceptible Tomato Genotypes

Leaf tissue was collected from symptomatic tomato plants in agricultural fields with TYLCV-resistant and -susceptible genotypes from Tifton, Georgia in 2015 and 2016 and from Immokalee, Florida, USA in 2015. Detailed sample information is included in Table 1. Whole genome sequences obtained from those leaf tissue samples were deposited in the GenBank with accession numbers KY971320–KY971372.

**Table 1:** Details of TYLCV isolates field-collected from susceptible and resistant tomato genotypes.

**Sample name**	**Collection date**	**Location**	**Host plant**	**TYLCV-susceptibility of host plant**	**Accession no**.	Resistant gene^*^
Florida_1.2	Mar-2015	USA: Immokalee, Florida	*Solanum lycopersicum* cv. FL47 (Seminis)	Susceptible	KY971320	None^#^
Florida_11.1_R	Apr-2015	USA: Immokalee, Florida	*Solanum lycopersicum* cv. Skyway 687 (Enza Zaden)	Resistant	KY971321	*Ty3Ty6^*$*^*
Florida_11.2_R	Apr-2015	USA: Immokalee, Florida	*Solanum lycopersicum* cv. Skyway 687 (Enza Zaden)	Resistant	KY971322	*Ty3Ty6*
Florida_11.3_R	Apr-2015	USA: Immokalee, Florida	*Solanum lycopersicum* cv. Skyway 687 (Enza Zaden)	Resistant	KY971323	*Ty3Ty6*
Florida_17.1	Jun-2015	USA: Immokalee, Florida	*Solanum lycopersicum* cv. Suddath's Strain (Nature and Nuture Seeds)	Susceptible	KY971324	None
Florida_18.1	Jun-2015	USA: Immokalee, Florida	*Solanum lycopersicum* cv. Orange Strawberry (Baker Creek Heirloom Seeds)	Susceptible	KY971325	None
Florida_19.1	Jun-2015	USA: Immokalee, Florida	*Solanum lycopersicum* cv. Legend (Tomato Growers)	Susceptible	KY971326	None
Florida_2.1	Apr-2015	USA: Immokalee, Florida	*Solanum lycopersicum* cv. Plum Regal (Bejo Seeds)	Susceptible	KY971327	None
Florida_3.1	Apr-2015	USA: Immokalee, Florida	*Solanum lycopersicum* cv. BHN 685 (Siegers Seed Company)	Susceptible	KY971328	None
Florida_4.2_R	Apr-2015	USA: Immokalee, Florida	*Solanum lycopersicum* cv. HM 8845 (Harris Moran Seed Company)	Resistant	KY971329	Unk.^∧^
Florida_4.4_R	Apr-2015	USA: Immokalee, Florida	*Solanum lycopersicum* cv. HM 8845 (Harris Moran Seed Company)	Resistant	KY971330	Unk.
Florida_5.1	Apr-2015	USA: Immokalee, Florida	*Solanum lycopersicum* cv. Juliet (Johnny's Selected Seeds)	Susceptible	KY971331	None
Florida_6.5	Apr-2015	USA: Immokalee, Florida	*Solanum lycopersicum* cv. Gator (Bejo Seeds)	Susceptible	KY971332	None
Florida_7.1	Apr-2015	USA: Immokalee, Florida	*Solanum lycopersicum* cv. yellow pear heirloom (Tomato Growers)	Susceptible	KY971333	None
Florida_7.2	Apr-2015	USA: Immokalee, Florida	*Solanum lycopersicum* cv. yellow pear heirloom (Tomato Growers)	Susceptible	KY971334	None
Florida_7.3	Apr-2015	USA: Immokalee, Florida	*Solanum lycopersicum* cv. yellow pear heirloom (Tomato Growers)	Susceptible	KY971335	None
Florida_7.5	Apr-2015	USA: Immokalee, Florida	*Solanum lycopersicum* cv. yellow pear heirloom (Tomato Growers)	Susceptible	KY971336	None
Florida_8.4	Apr-2015	USA: Immokalee, Florida	*Solanum lycopersicum* cv. Brickyard (Syngenta)	Susceptible	KY971337	None
Georgia_107.2	Sep-2016	USA: Tifton, Georgia	*Solanum lycopersicum* cv. Lanai (lab cultivar)	Susceptible	KY971338	None
Georgia_108.1	Sep-2016	USA: Tifton, Georgia	*Solanum lycopersicum* cv. FL47 (Seminis)	Susceptible	KY971339	None
Georgia_108.2	Sep-2016	USA: Tifton, Georgia	*Solanum lycopersicum* cv. FL47 (Seminis)	Susceptible	KY971340	None
Georgia_112.1	Sep-2016	USA: Tifton, Georgia	*Solanum lycopersicum* cv. FL47 (Seminis)	Susceptible	KY971341	None
Georgia_118.1_R	Sep-2016	USA: Tifton, Georgia	*Solanum lycopersicum* cv. Security (Harris Seeds Company)	Resistant	KY971342	*Ty1ty1*^&^
Georgia_122.2_R	Sep-2016	USA: Tifton, Georgia	*Solanum lycopersicum* cv. Security (Harris Seeds Company)	Resistant	KY971343	*Ty1ty1*
Georgia_124.3_R	Sep-2016	USA: Tifton, Georgia	*Solanum lycopersicum* cv. Inbar (Hazera Genetics)	Resistant	KY971344	*Ty1/3Ty6*^$^
Georgia_127.1_R	Sep-2016	USA: Tifton, Georgia	*Solanum lycopersicum* cv. Inbar (Hazera Genetics)	Resistant	KY971345	*Ty1/3Ty6*
Georgia_130.1_R	Sep-2016	USA: Tifton, Georgia	*Solanum lycopersicum* cv. Shanty (Hazera Genetics)	Resistant	KY971346	*Ty1*-^%^
Georgia_130.2_R	Sep-2016	USA: Tifton, Georgia	*Solanum lycopersicum* cv. Shanty (Hazera Genetics)	Resistant	KY971347	*Ty1-*
Georgia_132.1_R	Sep-2016	USA: Tifton, Georgia	*Solanum lycopersicum* cv. Shanty (Hazera Genetics)	Resistant	KY971348	*Ty1-*
Georgia_133.1_R	Sep-2016	USA: Tifton, Georgia	*Solanum lycopersicum* cv. Tygress (Seminis)	Resistant	KY971349	*Ty1ty1*^&^
Georgia_133.2_R	Sep-2016	USA: Tifton, Georgia	*Solanum lycopersicum* cv. Tygress (Seminis)	Resistant	KY971350	*Ty1ty1*
Georgia_135.1_R	Sep-2016	USA: Tifton, Georgia	*Solanum lycopersicum* cv. Tygress (Seminis)	Resistant	KY971351	*Ty1ty1*
Georgia_135.2_R	Sep-2016	USA: Tifton, Georgia	*Solanum lycopersicum* cv. Tygress (Seminis)	Resistant	KY971352	*Ty1ty1*
Georgia_25.1_R	Sep-2015	USA: Tifton, Georgia	*Solanum lycopersicum* cv. Security (Harris Seeds Company)	Resistant	KY971353	*Ty1ty1*
Georgia_30.1_R	Sep-2015	USA: Tifton, Georgia	*Solanum lycopersicum* cv. Shanty (Hazera Genetics)	Resistant	KY971354	*Ty1-*
Georgia_35.1	Sep-2015	USA: Tifton, Georgia	*Solanum lycopersicum* cv. FL47 (Seminis)	Susceptible	KY971355	None
Georgia_40.2_R	Oct-2015	USA: Tifton, Georgia	*Solanum lycopersicum* cv. Shanty (Hazera Genetics)	Resistant	KY971356	*Ty1-*
Georgia_47.1_R	Oct-2015	USA: Tifton, Georgia	*Solanum lycopersicum* cv. Tygress (Seminis)	Resistant	KY971357	*Ty1ty1*
Georgia_50.1_R	Oct-2015	USA: Tifton, Georgia	*Solanum lycopersicum* cv. Security (Harris Seeds Company)	Resistant	KY971358	*Ty1ty1*
Georgia_57.1	Oct-2015	USA: Tifton, Georgia	*Solanum lycopersicum* cv. FL47 (Seminis)	Susceptible	KY971359	None
Georgia_58.1	Oct-2015	USA: Tifton, Georgia	*Solanum lycopersicum* cv. Red Bounty (Harris Seeds Company)	Susceptible	KY971360	None
Georgia_58.2	Oct-2015	USA: Tifton, Georgia	*Solanum lycopersicum* cv. Red Bounty (Harris Seeds Company)	Susceptible	KY971361	None
Georgia_59.1	Oct-2015	USA: Tifton, Georgia	*Solanum lycopersicum* cv. FL47 (Seminis)	Susceptible	KY971362	None
Georgia_59.3	Oct-2015	USA: Tifton, Georgia	*Solanum lycopersicum* cv. FL47 (Seminis)	Susceptible	KY971363	None
Georgia_63.1_R	Oct-2015	USA: Tifton, Georgia	*Solanum lycopersicum* cv. Tygress (Seminis)	Resistant	KY971364	*Ty1ty1*
Georgia_67.2_R	Oct-2015	USA: Tifton, Georgia	*Solanum lycopersicum* cv. Tygress (Seminis)	Resistant	KY971365	*Ty1ty1*
Georgia_72.1_R	Oct-2015	USA: Tifton, Georgia	*Solanum lycopersicum* cv. Shanty (Hazera Genetics)	Resistant	KY971366	*Ty1-*
Georgia_76.2_R	Oct-2015	USA: Tifton, Georgia	*Solanum lycopersicum* cv. Security (Harris Seeds Company)	Resistant	KY971367	*Ty1ty1*
Georgia_81.3_R	Oct-2015	USA: Tifton, Georgia	*Solanum lycopersicum* cv. Security (Harris Seeds Company)	Resistant	KY971368	*Ty1ty1*
Georgia_9.10	Jan-2015	USA: Tifton, Georgia	*Solanum lycopersicum* cv. FL47 (Seminis)	Susceptible	KY971369	None
Georgia_9.9	Jan-2015	USA: Tifton, Georgia	*Solanum lycopersicum* cv. FL47 (Seminis)	Susceptible	KY971370	None
Georgia_92.1_R	Nov-2015	USA: Tifton, Georgia	*Solanum lycopersicum* cv. Security (Harris Seeds Company)	Resistant	KY971371	*Ty1ty1*
Georgia_99.1	Sep-2016	USA: Tifton, Georgia	*Solanum lycopersicum* cv. Lanai (lab cultivar)	Susceptible	KY971372	None

* *Heterozygous nature of Ty resistance genes in tomato cultivars and hybrids are indicated*.

# *None- implies that susceptible cultivars/hybrids did not carry a TYLCV resistant gene*.

∧ *Unknown- implies information not publicly available/experimentally determined yet*.

& *Ji et al. (2007)*.

$ *Genotyped for this study*.

% *Gelbart et al. (2020)*.

### Cloning and Sequencing of TYLCV Genomes

DNA from leaf tissue was extracted using GeneJET Plant Genomic DNA Purification Kit (Thermo Scientific, Waltham, MA). TYLCV DNA from susceptible tomato genotypes was amplified with rolling circle amplification. TYLCV DNA from resistant cultivars did not amplify optimally with rolling circle amplification, probably because resistant genotypes typically accumulated reduced levels of viral DNA than susceptible genotypes (Legarrea et al., 2015). Consequently, a PCR-based cloning method was employed to amplify TYLCV DNA from resistant genotypes. TYLCV DNA from susceptible genotypes was amplified using the TempliPhi (GE Healthcare, Chicago, IL) kit and the protocol outlined by Inoue-Nagata et al. (2004). Amplified DNA was digested with SacI (Fisher BioReagents, Pittsburgh, Pennsylvania). To purify the DNA, a gel extraction was performed on the SacI-digested DNA using crystal violet (Fisher Chemical, Fair Lawn, NJ) as the DNA-visualizing agent. The DNA was then ligated into the vector pGEM-3Z (Promega Corporation, Madison, WI) and a transformation was performed into One Shot TOP10 Chemically Competent *E. coli* (Invitrogen, Carlsbad, CA). Colonies were screened for TYLCV inserts via PCR with primers T7F (5′-TAATACGACTCACTATAGGG-3′) and M13R (5′- CAGGAAACAGCTATGACC-3′), and purified plasmids were sequenced (Eurofins Genomics, Louisville, KY) using the following primers: 5370F (5′-TTCGCTATTACGCCAGCT-3′), 2941R (5′-CCCAGGCTTTACACTTTATGCTTCC-3′), 710F (5′-TCTTATATCTGTTGTAAGGGCCCGT-3′), and 1400F (5′-ACGAGAACCATACTGAAAACGCCTT-3′).

TYLCV DNA from resistant genotypes was amplified using PCR with three different primer sets to cover the full-length of the TYLCV genome. The first segment was amplified with primers 1470R (5′-TGCATACACTGGATTAGAGGCATG-3′) and 2243F (5′-GAAACATAAACTTCTAAAGGAGGAC-3′), and a PCR program with an initial 95°C denaturation step for 3 min followed by 35 cycles of 95°C for 30s, 56°C for 30 s, and 72°C for 1 min, and a final extension step of 72°C for 5 min. The PCR mixture for each sample was 10 μl comprising 5 μl of GoTaq^®^ Green Master Mix (Promega Corporation, Madison, WI), 2 μl of water, 0.5 μl of each primer at 10 μM concentration, and 2 μl of DNA extract. The second segment was amplified with primers C2R (5′-CCAATAAGGCGTAAGCGTGT-3′) and 1371F (5′-AACTTATAATCATCAGGAGGCAGCC-3′), and the third segment was amplified with C2F (5′-GCAGTGATGAGTTCCCCTGT-3′) and 2326R (5′-GAGGCCCTCAATATATTAAAAGA-3′). Both the second and third segments were amplified with a PCR program with an initial denaturation step of 95°C for 3 min followed by 35 cycles of 95°C for 30 s, 55°C for 30 s, and 72°C for 50 s, and a final extension step of 72°C for 5 min. The three segments were cloned using CloneJET PCR Cloning Kit (Thermo Scientific, Waltham, MA). Ligated vectors were transformed into One Shot TOP10 Chemically Competent *E. coli* (Invitrogen, Carlsbad, CA). Colonies were screened via PCR to verify the ligation of expected-length inserts into plasmids. Five colonies that contained inserts of the proper length were chosen from each sample. Purified plasmids were then sequenced (Eurofins Genomics, Louisville, KY) using primers pJET1.2F (5′-CGACTCACTATAGGGAGAGCGGC-3′) and pJET1.2R (5′-AAGAACATCGATTTTCCATGGCAG-3′). Vector sequences were manually excised from TYLCV sequences, and the reads were assembled into full-length genomes using Geneious Pro v. 8.1.9 (Drummond et al., 2011).

### Whitefly-Mediated Serial Transmission of TYLCV to TYLCV-Resistant and -Susceptible Genotypes

Five tomato plants at the ten-leaf stage (~8 weeks old) of either the TYLCV-susceptible cultivar, Florida Lanai, or the TYLCV-resistant hybrid (*Ty1/3*-*Ty6*), Inbar (Hazera Genetics, Berurim M.P Shikmim, Israel), were individually caged in the greenhouse at the above-stated conditions. Viruliferous whiteflies were obtained by providing whiteflies with a 48 h acquisition access period (AAP) on TYLCV (KY965880)-infected tomato plant. The tomato plants were inoculated by clip-caging twenty viruliferous whiteflies to a fully expanded leaf at the upper portion of each plant. After a 48 h inoculation access period (IAP), the whiteflies were removed. The tomato plants were allowed to develop infection for 3 weeks and tested for TYLCV infection by PCR using the primers C2-1201 (5′- CATGATCCACTGCTCTGATTACA−3′) and C2-1800V2 (5′-TCATTGATGACGTAGACCCG-3′), which targeted a 695-nucleotide region of the TYLCV genome encompassing the entire C2 gene. The PCR mixture for each sample was 10 μl comprising 5 μl of GoTaq^®^ Green Master Mix, 2 μl of water, 0.5 μl of each primer at 10 μM concentration, and 2 μl of DNA extract. The PCR program had an initial denaturation step at 94°C for 2 min, followed by 30 cycles of 94°C for 30 s, 52°C for 30 s, and 72°C for 1 min, and a final extension at 72°C for 5 min. After 3 weeks, non-viruliferous whiteflies (20/plant) were then clip-caged to the upper leaves of TYLCV-infected plants (T_1_) and given a 48 h AAP. These whiteflies were subsequently transferred to non-infected plants of the same cultivar/hybrid (T_2_) for a 48 h IAP. The inoculated plants were maintained for 3 weeks, and TYLCV was again transmitted by whiteflies to non-infected plants. This process was repeated until whitefly-mediated transmission to tomato plants was completed 10 times (T_1_-T_10_). Leaf tissue samples were taken from each plant post first (T_1_), fifth (T_5_), and tenth (T_10_) transmission for DNA extraction. Three full-length TYLCV genomes were sequenced from all five replicates (plants) belonging to resistant and susceptible genotypes. Full-length genomes were cloned and sequenced as described earlier and deposited in GenBank (accession numbers KY965834 - KY965923).

### TYLCV Quantitation in Resistant and Susceptible Genotypes Following Whitefly-Mediated Serial Transmission

To assess if whitefly-mediated serial transmission over 10 passages affected TYLCV accumulation/TYLCV load differently in TYLCV-resistant genotypes and TYLCV-susceptible genotypes, DNA extracted from both susceptible and resistant genotypes at T_1_, T_5_, and T_10_ were subjected to absolute quantitation using real time PCR following the protocol outlined by Legarrea et al. (2015). Primers targeting a 102-bp region of the TYLCV C2 gene were used for this purpose (Legarrea et al., 2015). Plasmids with C2 gene inserts were used for generating a standard curve for absolute quantitation (Legarrea et al., 2015). DNA from 100 mg leaf tissue/plant corresponding to 10 to 15 resistant and susceptible genotypes at T_1_, T_5_, and T_10_ were used for absolute quantitation. A duplicate was included for all samples for the real time PCR runs. The virus copy numbers were analyzed using R Version 3.4.2 (R Core Team, 2019). Data were analyzed using a mixed-effect model in the “Lme4” package (Bates et al., 2015). Time intervals were considered as fixed effects and replications were as considered random effects. To meet the assumptions of normality and homoscedasticity of variance, virus copy numbers were log transformed. Differences in virus accumulation in susceptible and resistant genotypes leaf tissues independently were analyzed using one-way repeated-measures ANOVA, and treatment means were separated in the “emmeans” package with the default Tukey's honest significant difference (Tukey HSD) *post-hoc* test. To compare TYLCV copy numbers or virus loads in leaf tissues of resistant vs. susceptible genotypes a two-way analysis of variance was used. For this analyses, susceptibility/resistant status and transmission number were considered as fixed effects, replication was considered as random effect. Differences in virus loads between resistant and susceptible genotypes' leaf tissues at each transmission T_1_, T_5_, and T_10_ were assessed using the Tukey's honest significant difference (Tukey HSD) *post-hoc* test.

### Phylogenetic Analysis

A maximum-likelihood phylogenetic tree was constructed in MEGA X (Kumar et al., 2018). Fifty-three TYLCV genome sequences from samples collected from Florida and Georgia were used for phylogenetic analysis. Sequences were aligned in MUSCLE. The best-fitting nucleotide substitution model (Jukes-Cantor model) was determined by relying on the Akaike Information Criterion (AIC) in jMODELTEST (Darriba et al., 2012). The support for each individual branch was assessed via 1,000 bootstrap replications. For phylogenetic analyses involving comparison of genomes of Florida and Georgia isolates, a TYLCV genome sequence available from the GenBank (accession AY530931 from Florida) was also added to the data set. Representative genome sequences from each of the seven TYLCV strains viz., TYLCV-IL (GenBank accession number X15656), TYLCV-Bou (GenBank accession number GU076454), TYLCV-IR (GenBank accession number AJ132711), TYLCV-Kah (GenBank accession number EU635776), TYLCV-Ker (GenBank accession number GU076442), TYLCV-Mld (GenBank accession number X76319), and TYLCV-OM (GenBank accession number FJ956700) were included as outgroup taxa. Tomato yellow leaf curl China virus (TYLCCV) (GenBank accession number NC_004044) and TYLCSV (GenBank accession number GU951759) genome sequences were also included in the analyses.

### Nucleotide/Haplotype Diversity and Gene Flow and Genetic Differentiation

Nucleotide diversity (π), haplotype diversity, population mutation rate (θ), substitutions, and indels were calculated using the software DnaSP v5.10.01 (Librado and Rozas, 2009). To determine if TYLCV isolates obtained from TYLCV-resistant and -susceptible genotypes in Florida and/or Georgia differentiated from one another, nucleotide sequence-based Ks, Kst, Snn, Z, and Fst statistics were calculated using the Gene Flow and Genetic Differentiation tool in DnaSP (Hudson et al., 1992). To test for level of significance, a permutation test with 1,000 replications was performed. Values were considered significant if *p*-values were < 0.05.

### Positive Selection

All six genes of TYLCV genomes were analyzed for positive selection using the HyPhy tool (Pond and Muse, 2005) in MEGA 7.0.21 (Kumar et al., 2016). The HyPhy tool determined non-synonymous (dN) and synonymous (dS) nucleotide substitutions for each codon. The Tamura-Nei model was selected as the substitution model (Tamura and Nei, 1993). Codons with a dN greater than dS and a *p* < 0.05 were considered to be under positive selection.

### Population Neutrality

To test for neutrality among TYLCV isolates (populations) from TYLCV-resistant and susceptible genotypes, Tajima's D (Tajima, 1989) was computed in DnaSP using the Tajima's Test tool. Tajima's D statistic was determined by the average number of nucleotide pair-wise differences and the number of segregating sites among all sequences.

Fu and Li's D and F statistics (Fu and Li, 1993) were also calculated using DnaSP. The D statistic is calculated based on the number of mutations appearing just once and the total number of mutations. The F statistic is calculated based the number of mutations appearing just once and the average pairwise differences between sequences.

## Results

### TYLCV Isolates Field-Collected From TYLCV-Resistant and -Susceptible Genotypes

**Figure 2:**
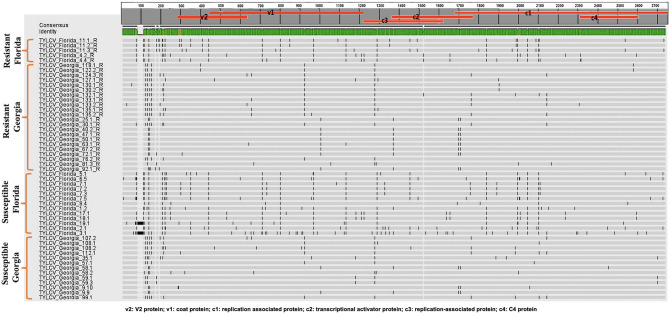
An alignment of full length TYLCV genomes corresponding to TYLCV-resistant and susceptible genotypes from Florida and Georgia with a single consensus sequence and mutations markings.

Twenty-seven TYLCV genomes from isolates of resistant genotypes and 26 TYLCV genomes from isolates of susceptible genotypes were sequenced and compared (Table 1). All TYLCV genomes isolated from resistant and susceptible genotypes were closely related to the TYLCV-IL strain than the other six TYLCV strains known. The nucleotide identities of isolated genomes ranged from of 96.61 to 98.25% in comparison with the TYLCV-IL strain genome (GenBank accession number X15656). The nucleotide identities of TYLCV genomes from resistant and susceptible genotypes in Florida ranged from 97.98 to 99.16%. The number of haplotypes were the same. However, the number of substitutions were higher in the TYLCV genomes isolated from susceptible than resistant Florida genotypes (Table 2). The nucleotide identities of TYLCV genomes from resistant and susceptible genotypes in Georgia ranged from 99.49 to 99.89%. The number of haplotypes was slightly higher in TYLCV genomes isolated from resistant genotypes than susceptible genotypes in Georgia. The number of substitutions and indels were also slightly higher in TYLCV genomes isolated from resistant genotypes than susceptible genotypes in Georgia (Table 2). The mutations in all genomes were more concentrated on the non-coding region (1–285 nucleotides) and on the viral strand genes (V1 and V2) than on the complementary strand genes (C1 through C4) (Figure 2).

**Table 2:** Nucleotide and haplotype diversity associated with TYLCV genomes isolated from resistant and susceptible tomato genotypes from Florida and Georgia.

**Populations analyzed**	**π^v^**	***θ^w^***	**Hap^x^**	**Substitutions^y^**	**Indel sites^z^**
Florida-R	0.012	0.014	3.00	91.00	3.00
Florida-S	0.011	0.017	3.00	148.00	3.00
Georgia-R	0.006	0.012	3.00	114.00	3.00
Georgia-S	0.007	0.012	2.00	102.00	2.00
Florida- R & S combined	0.011	0.019	4.00	156.00	6.00
Georgia- R & S combined	0.005	0.012	3.00	130.00	4.00

*R, Represents genomes from resistant genotypes*.

*S, Represents genomes from susceptible genotypes*.

v *π Represents nucleotide diversity*.

w *Population mutation rate*.

x *Number of haplotypes*.

y *Number of substitutions*.

z *InDel sites*.

*Reference sequence TYLCV-IL(X15656) was used in determining substitutions and indel sites*.

The average pairwise nucleotide differences (Kt) between genomes of TYLCV isolates from resistant vs. susceptible genotypes from Florida was 2.36 times higher than that from Georgia (Table 3). The nucleotide-based population differentiation statistics Ks, Kst, and Z values calculated were determined by the permutation test to be not significantly different between genomes of TYLCV isolates from resistant and susceptible genotypes from either Georgia or Florida (Table 3). On the contrary, the *p-*value indicated associated with another differentiation statistic Snn was significantly different between genomes of TYLCV isolates from resistant and susceptible Florida genotypes. However, the associated Fst between the genomes of TYLCV isolates from resistant and susceptible genotypes from Florida indicated a high level of similarity. When genomes of TYLCV isolates from resistant and susceptible genotypes from both states were combined, significant differences for Ks, Kst, Snn, and Z between were observed (Table 3). Overall, the Fst values were low when the genomes of TYLCV isolates from resistant and susceptible genotypes from Florida and/or Georgia were compared, indicating a high level of similarity between TYLCV isolates present in resistant and susceptible genotypes.

**Table 3:** Genetic differentiation statistics with genomes of TYLCV isolates field-collected from TYLCV-resistant and -susceptible tomato genotypes from Florida and Georgia.

**Populations analyzed**	**Kt^x^**	**Ks^y^**	**Kst^y^**	***p*-value of Ks and Kst**	**Snn^y^**	***p*-value of Snn**	**Z^y^**	***p*-value of Z**	**Fst^z^**
Florida_R vs. Florida_S	26.332	25.583	0.028	0.079	0.889	**0.011**	72.165	0.107	0.067
Georgia_R vs. Georgia_S	11.826	11.880	−0.004	0.487	0.588	0.193	299.427	0.577	−0.009
Resistant vs. Susceptible	24.620	23.861	0.031	**0.017**	0.680	**0.004**	670.422	**0.034**	0.059
Florida vs. Georgia	24.869	16.346	0.342	**0.000**	0.981	**0.000**	455.610	**0.000**	0.497

x *Kt is the average number of pairwise nucleotide differences across genomes in both populations*.

y *Ks, Kst, Snn, and Z are nucleotide sequence-based genetic differentiation statistics*.

z *Fst is a population differentiation statistic. Values range from 0 to 1. Low Fst values indicate a high level of similarity between populations while high Fst values indicate genetically distinct groups. Bold values indicate statistical significance*.

Positive selection on all six genes of Florida and Georgia TYLCV isolates from resistant and susceptible genotypes was assessed using the HyPhy codon selection test. No codon was determined to be under positive selection at a statistically significant level for any of the six genes (Supplementary Material 1).

Population neutrality statistics Fu and Li's D and F statistics and Tajima's D were calculated using the TYLCV genomes obtained from TYLCV-resistant and/or susceptible genotypes in Florida and/or Georgia. These statistics examined the frequency of segregating sites across the genome. Positive values for all three statistics were noticed in the case of genomes of TYLCV isolates from resistant genotypes from Florida, but the corresponding *p-*values indicated no statistical significance (Table 4). On the contrary, a significant *p-*value was accompanied by negative values for Fu and Li's D and F statistics in the case of genomes of TYLCV isolates from resistant genotypes in Georgia (Table 4). Negative values and insignificant *p-*values for all three statistics were obtained for genomes of TYLCV isolates from susceptible genotypes from either Florida or Georgia (Table 4). Similar results were obtained when genomes of TYLCV isolates from resistant genotypes from Florida and Georgia, and genomes of TYLCV isolates from susceptible genotypes from both Florida and Georgia were examined (Table 4). When the genomes of TYLCV isolates from resistant and susceptible genotypes from both states were combined, significant results for Fu and Li's F and D statistics were identified (Table 4). However, negative values for Fu and Li's D and F statistics reiterated evidence for either a recent population expansion or purifying selection.

**Table 4:** Tests of neutrality with genomes of TYLCV isolates field-collected from TYLCV-resistant and -susceptible tomato genotypes from Florida and Georgia.

**Population analyzed**	**Fu and Li's D^a^**	***p*-value**	**Fu and Li's F^a^**	***p*-value**	**Tajima's D^a^**	***p*-value**
Florida_R	0.177	*p* > 0.10	0.177	*p* > 0.10	0.279	*p* >0.10
Florida_S	−1.586	*p* > 0.10	−1.835	*p* > 0.10	−1.599	0.10> *p* > 0.05
Georgia_R	−2.527	***p*** **<** **0.05**	−2.55	***p*** **<** **0.05**	−1.053	*p* > 0.10
Georgia_S	−1.200	*p* > 0.10	−1.294	*p* > 0.10	−0.788	*p* > 0.10
Florida_R and Georgia_R	−1.186	*p* > 0.10	−1.32671	*p* > 0.10	−0.998	*p* > 0.10
Florida_S and Georgia_S	−2.158	0.10 > *p* > 0.05	−2.248	0.10 > *p* > 0.05	−1.383	*p* > 0.10
Florida	−1.92670	*p* > 0.10	−2.18758	0.10 > *p* > 0.05	−1.76221	0.10 > *p* > 0.05
Georgia	−2.14999	0.10 > *p* > 0.05	−2.12725	0.10 > *p* > 0.05	−1.10892	*p* > 0.10
Combined	−3.23971	***p*** **<** **0.05**	−3.15494	***p*** **<** **0.05**	−1.66634	0.10 > *p* > 0.05

a *Negative Fu and Li's D and F values and Tajima's D values indicate population expansion or purifying selection. Bold values indicate statistical significance*.

### TYLCV Isolates Field-Collected From Florida and Georgia

The genomes of TYLCV isolates were divided based on the state they were collected from and regardless of the genotype resistance status. The nucleotide identities for TYLCV genomes between Florida and Georgia isolates ranged from 98.62 to 99.20%. The number of haplotypes were higher in TYLCV genomes isolated from Florida than Georgia regardless of resistant status of genotypes. The number of substitutions and indels were also higher in TYLCV genomes isolated from Florida than Georgia regardless of resistant status of genotypes (Table 2). The mutations in all genomes were more concentrated on the non-coding region (1–285 nucleotides) and on the viral strand (V1 and V2) genes than on the complementary strand genes (C1 through C4) (Figure 2).

The nucleotide-based genetic differentiation statistics Ks, Kst, Snn, and Z statistics with their corresponding permutation tests revealed differences between two populations (Table 3).

All six genes from the Florida, Georgia, and combined populations were tested with the HyPhy codon selection test. No codon was under positive selection at a statistically significant level (Supplementary Material 1).

The genomes of Florida and Georgia isolates regardless of the genotype resistance status were tested for neutrality with Fu and Li's D and F and Tajima's D statistics. The Florida and Georgia populations both had negative values for all three statistics, and they were not at a statistically significant level (Table 4). The combined population had statistically significant values for Fu and Li's D and F statistics but not for Tajima's D. The negative values of the Fu and Li's D and F statistics once again indicated evidence for either population expansion or purifying selection.

**Figure 3:**
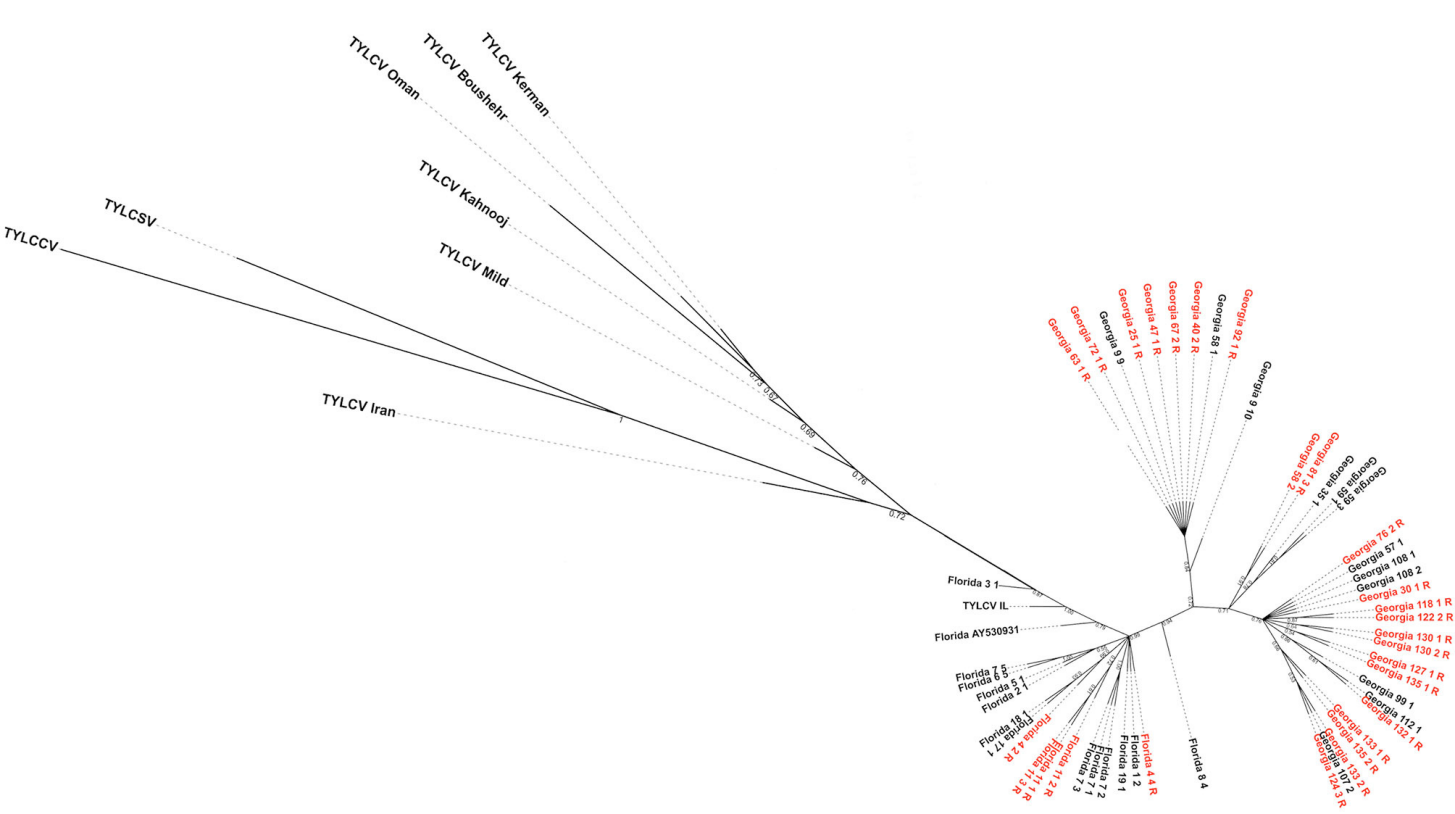
Maximum-likelihood phylogenetic tree constructed with field-collected TYLCV genomes from Florida and Georgia. Samples with a “R” at the end of their name and labeled in red were isolated from a resistant genotype. All other TYLCV genomes were isolated from a susceptible genotype, except “Florida GenBank accession# AY530931” whose TYLCV susceptibility status is unknown. Representative genome sequences from each of the seven TYLCV strains viz., TYLCV-IL, TYLCV-Boushehr, TYLCV-Iran, TYLCV-Kahnooj, TYLCV-Kerman, TYLCV-Mild, and TYLCV-Oman were included as outgroup taxa. Tomato yellow leaf curl China virus and tomato yellow leaf curl Sardinia virus genome sequences were also included in the analyses.

The maximum likelihood tree showed the Florida and Georgia TYLCV isolates clearly parsed from one another into separate clades (Figure 3). The Georgia clade/s appeared to emerge from the Florida population. This could indicate that the Georgia TYLCV populations arose from an introduction into Florida. The TYLCV samples from resistant and susceptible genotypes did not parse with one another. There does not appear to be any phylogenetic relationship between TYLCV genomes and the resistance status of the genotypes they were collected from.

### Whitefly-Mediated Serial Transmission of TYLCV to TYLCV-Resistant and -Susceptible Genotypes

**Figure 4:**
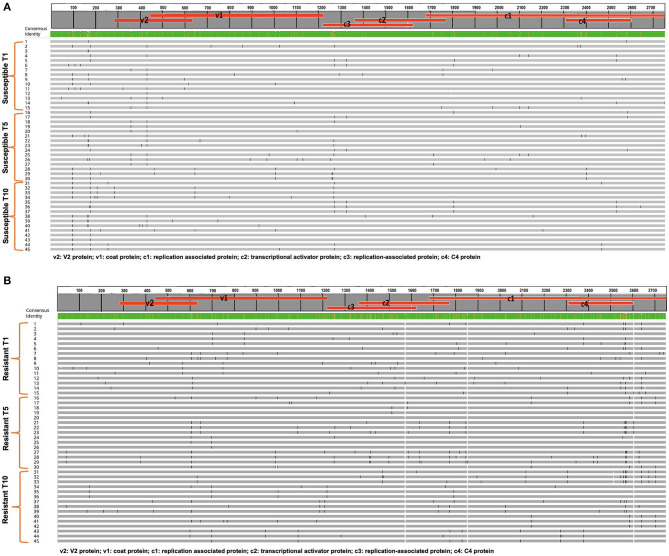
**(A)** An alignment of full length TYLCV genomes corresponding to the TYLCV-susceptible genotype Lanai following whitefly mediated serial transmission. Fifteen genomes from transmission 1 (T1), transmission 5 (T5), and transmission (10) are included along with a consensus sequence. Mutations in each genome are tracked. **(B)** An alignment of full length TYLCV genomes corresponding to the TYLCV-resistant genotype Inbar following whitefly mediated serial transmission. Fifteen genomes from transmission 1 (T1), transmission 5 (T5), and transmission (10) are included along with a consensus sequence. Mutations in each genome are tracked.

TYLCV was successfully serially transmitted 10 times (T_1_-T_10_) via whiteflies to both TYLCV-resistant and susceptible genotypes. TYLCV genomes from both resistant and/or susceptible genotypes at T_1_, T_5_, and T_10_ were assessed to determine if TYLCV populations from the susceptible and/or resistant genotypes differentiated from one another. The nucleotide identities of TYLCV genomes isolated from resistant and susceptible genotypes following serial transfer ranged from 99.35 to 99.75%. The number of haplotypes were higher in TYLCV genomes isolated from the resistant genotype than the susceptible genotype (Table 5). The number of substitutions and indels were also higher in TYLCV genomes isolated from the resistant genotype than the susceptible genotype (Table 5). The mutations were more concentrated on the non-coding region (1–285 nucleotides) and on the viral strand (V1 and V2) genes than on the complementary strand genes (C1 through C4) (Figures 4A,B).

**Table 5:** Nucleotide and haplotype diversity associated with TYLCV genomes isolated from a TYLCV-resistant and susceptible tomato genotype following serial transmission of TYLCV.

**Populations analyzed**	**π^v^**	***θ^w^***	**Hap^x^**	**Substitutions^y^**	**Indel sites^z^**
Serial transmission R T_1_-T_10_	0.007	0.021	8.00	229.00	8.00
Serial transmission S T_1_-T_10_	0.004	0.012	4.00	142.00	4.00

*R, Represents genomes from the resistant genotype following 10 serial transmissions (T_1_-T_10_)*.

*S, Represents genomes from susceptible genotypes*.

v *π Represents nucleotide diversity*.

w *Population mutation rate*.

x *Number of haplotypes*.

y *Number of substitutions*.

z *InDel sites*.

*Reference sequence TYLCV-IL(X15656) was used in determining substitutions and indel sites*.

With the TYLCV susceptible genotype, the nucleotide sequence-based genetic differentiation statistics Ks, Kst, Snn, and Z showed a statistically significant differentiation occurring at T_5_, and T_10_, but not T_1_ (Table 6). But the Fst values were low indicating lack of differentiation with increasing transmission number. Similar results were also obtained with the resistant genotype, indicating no evidence of population differentiation with increasing transmission number (Table 6). When the TYLCV genomes from the resistant genotype were compared with TYLCV genomes from susceptible genotype, genetic differentiation statistics Ks, Kst, Snn, and Z were statistically significant at T_5_, and T_10_, but not T_1_ (Table 6). Nevertheless, the Fst values were still low and indicated no genetic differentiation in TYLCV genomes between resistant and susceptible genotypes following serial transfer.

**Table 6:** Genetic differentiation statistics with genomes of TYLCV isolates from a TYLCV-resistant and -susceptible genotype at different stages of whitefly-mediated serial transmission.

**Populations analyzed**	**Kt^x^**	**Ks^y^**	**Kst^y^**	***p*-value of Ks and Kst**	**Snn^y^**	***p*-value of Snn**	**Z^y^**	***p*-value of Z**	**Fst^z^**
S1 vs. S5	8.718	8.685	0.003	0.310	0.712	**0.005**	216.623	0.395	0.007
S1 vs. S10	8.595	8.076	0.060	**0.001**	0.827	**0.000**	196.785	**0.001**	0.110
S5 vs. S10	8.834	8.400	0.049	**0.001**	0.900	**0.000**	203.333	**0.005**	0.090
R1 vs. R5	15.480	14.971	0.032	**0.007**	0.600	0.126	215.297	0.250	0.061
R1 vs. R10	15.790	15.428	0.022	**0.014**	0.813	**0.000**	210.557	0.055	0.043
R5 vs. R10	15.082	14.647	0.028	**0.024**	0.833	**0.000**	212.542	0.108	0.054
R1 vs. S1	11.820	11.790	0.002	0.331	0.467	0.640	214.121	0.161	0.005
R5 vs. S5	12.071	11.400	0.055	**0.001**	0.933	**0.000**	202.683	**0.002**	0.102
R10 vs. S10	12.429	11.380	0.084	**0.000**	0.933	**0.000**	188.652	**0.000**	0.151

x *Kt is the average number of pairwise nucleotide differences across genomes in both populations*.

y *Ks, Kst, Snn, and Z are nucleotide sequence-based genetic differentiation statistics*.

z *Fst is a genetic differentiation statistic. Values range from 0 to 1. Low Fst values indicate a high-level similarity between populations while high Fst values indicate genetically distinct groups. Bold values indicate statistical significance*.

All six genes were tested for positive selection by determining the non-synonymous to synonymous substitution ratio (dN/dS) for each codon. TYLCV genome sequences from resistant and susceptible genotypes were tested both separately and together at T_1_, T_5_, and T_10_. No statistically significant (*p* < 0.05) positive selection of any of the codons was detected (Supplementary Material 1).

Fu and Li's F and D statistics and Tajima's D were calculated for TYLCV genomes from TYLCV- resistant and -susceptible genotypes at T_1_, T_5_, and T_10_. Fu and Li's F and D statistics and Tajima's D were only significant for the genomes from the resistant genotype at T_1_ (Table 7). However, Fu and Li's F and D statistics and Tajima's D were all negative for TYLCV genomes from both susceptible and resistant genotypes at T_1_, T_5_, and T_10_ (Table 7)_._ Again, these statistics provided evidence for purifying selection and/or population expansion than positive selection.

**Table 7:** Tests of neutrality with genomes of TYLCV isolates from a TYLCV-resistant and -susceptible genotype at different stages of whitefly-mediated serial transmission.

**Population analyzed**	**Fu and Li's D^a^**	***p*-value**	**Fu and Li's F^a^**	***p*-value**	**Tajima's D^a^**	***p*-value**
Transmission 1 (T_1_)-Resistant	−2.39208	***p*** **<** **0.05**	−2.59396	***p*** **<** **0.05**	−1.88472	***p*** **<** **0.05**
Transmission 5 (T_5_)-Resistant	−0.63476	*p* > 0.10	−0.78389	*p* > 0.10	−0.79782	*p* > 0.10
Transmission 10 (T_10_)-Resistant	−0.91053	*p* > 0.10	−1.12974	*p* > 0.10	−1.15968	*p* > 0.10
Transmission 1 (T_1_)-Susceptible	−1.22645	*p* > 0.10	−1.45762	*p* > 0.10	−1.36701	*p* > 0.10
Transmission 5 (T_5_)-Susceptible	−1.10155	*p* > 0.10	−1.28177	*p* > 0.10	−1.14230	*p* > 0.10
Transmission 10 (T_10_)-Susceptible	−1.06583	*p* > 0.10	−1.20065	*p* > 0.10	−0.98299	*p* > 0.10

a *Negative Fu and Li's D and F values and Tajima's D values indicate population expansion or purifying selection. Bold values indicate statistical significance*.

### TYLCV Quantitation in Resistant and Susceptible Genotypes Following Whitefly-Mediated Serial Transmission

**Figure 5:**
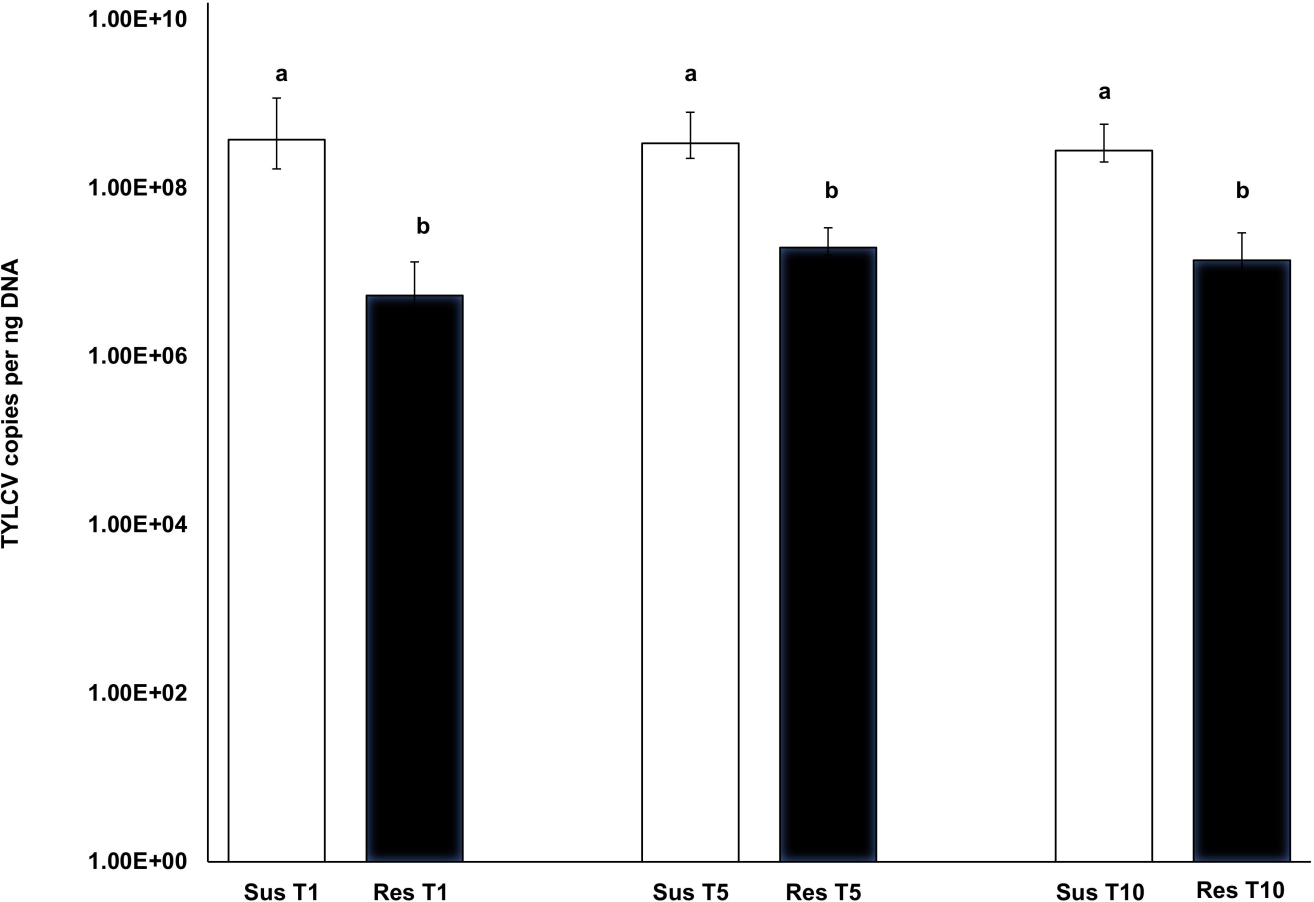
Absolute quantitation of TYLCV loads in the susceptible genotype (Lanai) and resistant genotype (Inbar) following serial transmission at T_1_, T_5_, and T_10_. DNA extracted from both susceptible and resistant genotypes at T_1_, T_5_, and T_10_ were subjected to absolute quantitation using real time PCR. Differences in virus loads between resistant and susceptible genotypes' leaf tissues at each transmission T_1_, T_5_, and T_10_ were assessed using the Tukey's honest significant difference (Tukey HSD) *post-hoc* test. Differences in mean separation letters indicate differences in TYLCV loads between the susceptible and resistant genotypes at each serial transfer.

TYLCV-induced symptoms were more prominent in the susceptible genotype and quite subdued in the resistant genotype. The symptom severity did not change in either the susceptible or resistant genotype with increasing transmission number. TYLCV virus loads from leaf tissue corresponding to resistant genotype plants did not differ with transmission number [T_1_-T_5_: *F*_(1, 28)_ = 2.01, *p* = 0.337; T_1_-T_10_: *F*_(1, 25)_ = 0.44, *p* = 0.997; T_5_-T_10_: *F*_(1, 25)_ = 1.51, *p* = 0.624] (Figure 5). Similarly, TYLCV virus loads from leaf tissue corresponding to susceptible genotype plants did not differ with transmission number [T_1_-T_5_: *F*_(1, 27)_ = 0.13, *p*= 0.999; T_1_-T_10_: *F*_(1, 23)_ = 1.160, *p* = 0.855; T_5_-T_10_: *F*_(1, 23)_ = −1.291, *p* = 0.790] (Figure 5). However, TYLCV loads between susceptible and resistant plants varied significantly at T_1_ [*F*_(1, 28)_ = 5.23, *p* < 0.001], T_5_ [*F*_(1, 27)_ = 3.42, *p* = 0.004] and T_10_ [*F*_(1, 20)_ = 6.14, *p* < 0.001] (Figure 5). TYLCV loads were higher in the susceptible genotype than in the resistant genotype at T_1_, T_5_, and T_10_.

## Discussion

High incidences of TYLCV are becoming the norm in the southeastern United States and in many other tomato-producing parts of the world. TYLCV-resistant genotypes are considered as the most effective management tool in the fight against TYLCV and are increasingly being used. This study attempted to assess whether the increased use of TYLCV-resistant genotypes can lead to selection pressure on the virus. Also, with multiple tomato crops grown in a calendar year in many locations, continuous planting of resistant genotypes could exert selection pressure on the virus. Positive selection against the virus could in turn lead to development of resistance-breaking strains of TYLCV. However, results in this study provided scant evidence to suggest that TYLCV is currently facing positive selection pressure stemming from the use of TYLCV-resistant genotypes in Florida and Georgia. Nevertheless, genetic differentiation was observed between TYLCV populations found in Florida and Georgia. The serial transmission assays also did not provide evidence for positive selection against TYLCV after 10 transfers (T_1_-T_10_). Overall, the TYLCV populations examined in this study seem to be shaped by purifying selection and/or population expansion than positive selection.

The genome sequences obtained from TYLCV-resistant and susceptible genotypes in both states were >97.5% similar in nucleotide identity. The phylogenetic analysis reveals that all the genome sequences from this study were closely related to the TYLCV-IL strain. The begomovirus strain demarcation limit is 94% nucleotide identity, and seven different TYLCV strains have been recognized thus far (Brown et al., 2015). TYLCV-IL seems to be only predominant strain in the southeastern United States despite evidence for multiple introductions of TYLCV (Duffy and Holmes, 2007). In spite of the high nucleotide sequence similarity among the genomes sequenced in this study, the phylogenetic analysis indicated that the Florida and Georgia samples parsed out into distinct clades. The phylogenetic tree hinted that the Georgia TYLCV population may be derived from an introduction from the Florida population. TYLCV was initially introduced into the Southeastern United States in Florida in 1996 or 1997 (Polston et al., 1999). In 1998, TYLCV was reported from South Georgia (Momol et al., 1999), therefore it seems likely that the Georgia population originated from a northern spread of the TYLCV population that first entered Florida. The absence of evidence of selection forces both in Florida and in Georgia suggests that the observed genetic differentiation between the two populations is shaped by population expansion and/or purifying selection aided by differences in introduction history, host availability, weather patterns, and agricultural practices that occur between the two states.

Mutations in TYLCV genomes predominantly included substitutions and fewer insertions and/or deletions. Mutations were at times higher in resistant genotypes and were generally higher in all genotypes from Florida. The Increased mutations from resistant genotypes than susceptible genotypes should be cautiously interpreted, as the genomes were sequenced using three sets of primers as opposed to RCA. This exercise could have inadvertently accounted for some of the observed mutations. However, no genetic differentiation was observed between genomes isolated from susceptible and resistant genotypes. The lack of significant results from the hypothesis testing using phylogenies (HyPhy) suggested absence of adaptive evolution or significant positive selection currently acting at the codon level. Positive selection pressure has been responsible for resistance breakdown against several viruses such as BNYVV and TSWV (Roggero et al., 2002; Aramburu and Marti, 2003; Ciuffo et al., 2005; Bornemann et al., 2015; Ferrand et al., 2015; Jiang et al., 2016; Batuman et al., 2017). The results in this study reiterated that the TYLCV populations in resistant and susceptible genotypes are being shaped by purifying selection and/or population expansion. There are several differences between *Ty*-mediated resistance and other dominant gene conferred resistance. Unlike the hypersensitive response observed in the case of *Sw5* and *Tsw* governed resistance against TSWV in tomato and pepper, respectively, *Ty*-mediated resistance results in systemic infection of the plant with mild to moderate symptoms and virus accumulation is typically at a reduced level than susceptible genotypes (Lapidot et al., 2001; Legarrea et al., 2015). Similar results were observed with TSWV-resistant peanut cultivars, which do not exhibit hypersensitive response, get systemically infected, and display mild to moderate symptoms upon infection (Shrestha et al., 2013; Sundaraj et al., 2014). Positive selection pressure was not observed in the case of TSWV-resistant peanut genotypes either (Sundaraj et al., 2014). The *Ty*1-6 resistance conferring genes vary in their biochemistry and differ in their mode of action (Ji et al., 2009; Verlaan et al., 2013; Lapidot et al., 2015; Yamaguchi et al., 2018; Gill et al., 2019). Of all the *Ty* gene combinations in the sampled genotypes, Ty1 seems to be the most common. The Ty1 gene belongs to the plant class of RNA dependent RNA polymerase (RDRPγ type) (Verlaan et al., 2013). Plant RDRPs are capable of targeting uncommon RNA molecules such as viruses and silencing them through the RNA interference mechanism (Ahlquist, 2002; Schwach et al., 2005; Díaz-Pendón et al., 2010; Garcia-Ruiz et al., 2010). In addition, many resistant genotypes seem to contain more than one *Ty* gene. These factors together could be contributing to reduction in selection pressure against the virus.

The whitefly-mediated TYLCV serial transmission experiment conducted to simulate continuous exposure of TYLCV, indicated that genetic differentiation in TYLCV genomes did not increase with serial transmission number, and was not different between the resistant and susceptible genotype. The dN/dS ratios calculated by the HyPhy codon selection test did not detect positive selection on any of the codons in the six TYLCV genes from genomes of the resistant or the susceptible genotype (Supplementary Material 1). The serial transmission experiment in this study lasted ~210 days. It is possible that with more time, the resistant and susceptible populations might have further differentiated, and positive selection may have eventually occurred. With another begomovirus, tomato yellow leaf curl China virus (TYLCCV), Ge et al. (2007) observed variation in the population structure following natural inoculation in tomato and experimental TYLCCV clone inoculation in *Nicotiana benthamiana* Domin. plants. Mutations in TYLCCV genomes did not vary between 60 and 120-days post inoculation. The observed mutations did not deviate much from progenitor sequences with a mutation rate of ~10^−4^, suggesting that TYLCCV was resembling a quasispecies and its mutation rate was similar to an RNA virus aided by purifying selection and population differentiation (Ge et al., 2007). Ge et al. (2007) also stated that TYLCCV mutation was responsible for its diversification, but it was somehow constrained. Similarly, in the current study, there seems to be evidence for the quasispecies nature of TYLCV and population diversification in general regardless of the susceptibility status of the host genotype or the geography. The lack of positive selection and hot spots in genomes analyzed in this study also point to constrained diversity driven by purifying selection and/or population expansion. Of course, the caveats in this study pertaining to the sampling size and sampling locations deserve further scrutiny. Another reason for the lack of significant population differentiation and/or selection could include the fact that the resistant genotype (Inbar) had multiple resistant genes viz., *Ty-*1/3 and *Ty-*6 with ability to confer at least two modes of resistance. In addition, all TYLCV genomes from Inbar were assembled using three PCR primer sets, this could have inadvertently accounted for an artifactual increase of substitutions and/or indels. Inadvertent introduction of mutations could be influenced by the presence of multiple isolates in the inoculum source. However, the original inoculum source used in this study was a susceptible genotype (Lanai), and RCA amplifications from that genotype only revealed the presence of a single isolate. Therefore, it is possible that the mutations observed in the resistant genotype Inbar may not be artificially introduced. The TYLCV-susceptible (Lanai) and TYLCV resistant (Inbar) are not near isogenic lines, the innate differences in their genetic background, besides *Ty* genes, could have also influenced the increased mutations in the resistant genotype. TYLCV resistant genotypes typically display less severe symptoms than susceptible genotypes, and they accumulate less virus than susceptible cultivars (Lapidot et al., 2001; Legarrea et al., 2015). Virus symptoms were less severe in the resistant genotype Inbar and did not change with serial transmission. The virus loads, as determined by qPCR in this study, was lower in the resistant genotype than the susceptible genotype at T_1_, T_5_, and T_10_. Virus loads also did not increase with transmission number in either the susceptible or resistant genotype. There was no evidence of development of a highly virulent or resistance-breaking strain characterized by enhanced symptom severity and/or increased virus load following serial transmission in the resistant genotype.

Recombination can play a major role in the evolution of begomoviruses (Navas-Castillo et al., 2000; García-Andrés et al., 2007; Moriones et al., 2007; Belabess et al., 2016). Recombinants of begomovirus species occur in nature especially aided by mixed infection and recombinants could also increase in frequency with time (García-Andrés et al., 2007). Resulting recombinants could produce a phenotype in infected hosts that is more pathogenic than the parental strains/species as seen in the case of TYLCSV and TYLCV in Spain (Monci et al., 2002). Also, begomovirus recombinants can outcompete parental virus strains in resistant cultivars as seen in southern Morocco and produce a more severe phenotype than parental virus strains (Belabess et al., 2016). It is possible that recombination could be occurring among TYLCV isolates used in this study, but the genomes of TYLCV isolates were very similar to one another within the two geographic regions and between the TYLCV-resistant and -susceptible genotypes, therefore making it impossible to detect recombination. Another tomato-infecting begomovirus, tomato mottle virus (ToMoV), is present in Florida, but it has not been recorded in Georgia (Akad et al., 2007). TYLCV is monopartite, whereas ToMoV is bipartite. TYLCV and ToMoV have been documented to co-infect individual tomato plants in Florida (Akad et al., 2007), but there is no indication that these two viruses could recombine. Elsewhere, TYLCV has been documented to recombine with several monopartite begomoviruses (Bananej et al., 2004; Idris and Brown, 2005; Guo et al., 2009; Kim et al., 2011; Park et al., 2011; Urbino et al., 2013). Introduction of a new begomovirus, specifically a monopartite species or a different strain of TYLCV, could offer more opportunities for recombination in the southeastern United States. Seven different strains of TYLCV have been identified thus far worldwide (Brown et al., 2015). In the southeastern United States, as shown in this study, TYLCV-IL seems to be only strain. Nevertheless, that scenario could change.

The availability of TYLCV-resistant cultivars/hybrids with improved horticultural traits and substantial whitefly pressure becoming the pattern, resistant genotypes have become a rather obvious choice for tomato growers in the southeastern United States. Currently, TYLCV resistant genotypes are planted in ~40% of the production acreage in Florida and Georgia (Ozores-Hampton et al., 2010; Srinivasan et al., 2012; Riley and Srinivasan, 2019). Based on information obtained in this study, the use of TYLCV-resistant tomato genotypes has not led to the development of resistance-breaking strains. However, positive selection and/or recombination with newly introduced TYLCV strains could change this scenario. Certain cropping strategies can be employed to reduce the risks of emergence of resistance-breaking strains (Fabre et al., 2012). One strategy is to plant a mixture of resistant and susceptible genotypes of tomato in order to reduce the overall selection pressure on the virus from the resistant genotype. This might already be unwillingly happening in the southeastern United States. The other strategy is to plant only resistant varieties on a landscape level. This strategy could help reduce the overall inoculum level in the landscape over time, as resistant genotypes typically accumulate less virus than susceptible genotypes (Lapidot et al., 2001; Legarrea et al., 2015). Resistant cultivars are an invaluable tool for growing tomatoes in TYLCV-affected areas and measures should be taken to preserve their usefulness.

## Conclusion

Virus-host interactions influenced by resistance-conferring dominant genes in several instances have placed substantial selection pressure on viruses. The resultant evolution of resistance-breaking strains has jeopardized the usefulness of resistant genotypes, wherein in many instances, rendering the only viable management option ineffective. With increasing whitefly and virus pressure in many tomato growing areas worldwide, reliance on TYLCV-resistant cultivars/hybrids is rising. Nevertheless, implications of usage of resistant genotypes under field conditions on rapid evolution of highly virulent or resistance-breaking TYLCV strains have been sparsely explored. This study made a preliminary attempt to examine the possibility of evolution of hot spots in the virus genome isolated from resistant genotypes that could trigger evolution of resistance-breaking strains. The lack of hypersensitive response to TYLCV as in the case of infection of several RNA viruses and the permissive replication due to systemic infection of TYLCV in resistant genotypes could be pivotal in preventing positive selection. However, introduction of other TYLCV strains and ensuing recombination events could alter that scenario. The TYLCV population structure in the southeastern United States at this moment seems to be determined by purifying selection and/or population expansion despite the use of resistant genotypes. Adoption of risk reduction strategies as outlined above could limit the development of resistance-breaking strains and facilitate the sustainable long-term usage of TYLCV-resistant genotypes.

## Data Availability Statement

The datasets presented in this study can be found in online repositories. The names of the repository/repositories and accession number(s) can be found in the article/Supplementary Material.

## Author Contributions

WM and RS designed the experiments. WM conducted the experiments and prepared the original draft of the manuscript. RS supervised the project. WM, SH, and SG performed the data analyses. All authors reviewed and approved the final version of the manuscript.

## Conflict of Interest

The authors declare that the research was conducted in the absence of any commercial or financial relationships that could be construed as a potential conflict of interest.
